# Temporomandibular dysfunction experience is associated with oral health-related quality of life: an Australian national study

**DOI:** 10.1186/s12903-021-01773-z

**Published:** 2021-09-06

**Authors:** Kamal Hanna, Rahul Nair, Najith Amarasena, Jason M. Armfield, David S. Brennan

**Affiliations:** 1 Australian Research Centre for Population Oral Health (ARCPOH), Adelaide Dental School, Faculty of Health and Medical Sciences, The University of Adelaide, Level 9 AHMS, Adelaide, SA 5005 Australia; 2 Quality and Safety of Oral Health Care Radboud UMC, Nijmegen, Netherlands; 3 Australian Centre for Precision Health, University of South Australia, Adelaide, Australia

## Abstract

**Background:**

There are very few studies of the association between temporomandibular dysfunction (TMD) and oral health-related quality of life (OHRQoL) in a representative sample from the Asia–Pacific region. Accordingly, we aimed to quantify the association of TMD with OHRQoL dimensions and overall measurement scores in a representative sample of Australian adults while accounting for a range of confounders, and statistically estimating whether TMD experience is meaningfully associated with OHRQoL.

**Method:**

Australia’s National Survey of Adult Oral Health 2004–2006 data were used. The outcome variables were the Oral Health Impact Profile (OHIP-14) domains and overall scores while the main exposure was self-reported Diagnostic Criteria Question for TMD. The analysis accounted for confounders including oral health status obtained from the oral examination, demographics, socioeconomics, health behaviours and health including perceived stress subscales of the PSS-14. We conducted complex samples analysis while using Cohen’s *f*^2^ effect size to estimate whether the association is meaningful.

**Results:**

TMD prevalence was 9.9% (95% CI: 8.4–11.6%) among 4133 Australian adults. TMD experience was associated with impairments to the seven OHIP-14 OHRQoL domains (*P* < .05) with higher impairments observed in physical pain (*B* = 0.82, 95% CI: .45–1.20, *P* < .001), psychological discomfort (*B* = 0.68, 95% CI: .29–1.06, *P* = .001) and psychological disability (*B* = 0.52, 95% CI: .20–.84, *P* = .001) in adjusted multivariate analyses. The difference in the mean OHIP-14 scores for those reporting TMD (Mean = 13.1, 95% CI: 12.0–14.0) and those who did not (Mean = 6.6, 95% CI: 6.0–6.8) was significant (*t* = 7.51, *P* < .001). In an adjusted multivariable model for OHIP-14 scores, TMD experience was associated with higher OHIP-14 scores (*B* = 3.34, 95% CI: 1.94–4.75, *P* < .001) where the Cohen’s *f*^*2*^ was .022. Further, perceived stress subscales: perceived distress and perceived control were associated with TMD experience and OHIP-14 scores (*P* < .05).

**Conclusion:**

Lower OHRQoL was observed in Australian adults who reported TMD experience but with small clinical importance which might support considering TMD in regular dental care. The higher impairments observed in physical pain, psychological discomfort and psychological disability domains of OHRQL can help clinicians and researchers focus their attention on these domains. The confounding effect exhibited by the perceived stress subscale might support their inclusion in future TMD and OHRQoL research.

**Supplementary Information:**

The online version contains supplementary material available at 10.1186/s12903-021-01773-z.

## Introduction

Temporomandibular dysfunction (TMD) is a group of degenerative musculoskeletal disorders that affects the morphology and function of the masticatory system [1]. In Australia, TMD is experienced by almost 10% of Australian adults [1] where evidence suggests that TMD symptoms might be negatively associated with individual’s perception of their physical, mental and social wellbeing about oral health [2–4] which is known as oral health-related quality of life (OHRQoL) [5]. While associations between components of oral health status such as caries experience and periodontal diseases, and OHRQoL have been investigated among the Australian adult population [6, 7], there is a lack of assessment of the association between TMD experience and OHRQoL among the Australian adult population. Understanding the OHRQoL impairment associated with TMD experience might be important for oral health education and treatment-seeking considering a minority of TMD patients (3–7%) are seeking professional advice [8]. While OHRQoL is known to be a multidimensional concept [5], there are limitations for studies from Australia that assessed which OHRQoL dimensions might be impaired by TMD experience.

TMD experience might be associated with impairment to several dimensions of OHRQoL. For instance, orofacial pain is the most common symptom experienced by TMD patients which severely affects the individual’s QoL [3, 9]. Furthermore, it is observed in another oral health condition that severe pain might interact with other QoL aspects such as mood status and the ability to perform daily activities such as work or study [10]. Besides, TMD patients experience chewing difficulty which is known to be negatively associated with OHRQoL [11]. This chewing difficulty often results in a dietary modification to reduce symptoms. The dietary modifications also constitute part of the conservative management plans for TMD [12], which is negatively associated with food enjoyment—an important OHRQoL aspect. Moreover, TMD patients might experience an audible click or grating [12] which might affect the social life of TMD patients. Understanding TMD association with dimensions and the overall OHRQoL among a representative sample of the Australian adult population might be important for clinical practice and health services research considering that researchers who conducted systematic reviews of the association between TMD and OHRQoL did not include representative population-based samples [2, 3]. Beyond investigating the association of TMD experience with OHRQoL, it is becoming necessary to capture whether TMD experience has a clinically relevant association with the OHRQoL measurement-a concept known as the Minimally Important Difference (MID).

There is a growing demand to estimate the Minimally Important Difference (MID) in measures of OHRQoL between different groups based on their disease status in the population [13]. Determining the clinical relevance of the association between TMD experience and OHRQoL is important for decision-making on whether a treatment is needed as well as to choose the intervention to be provided (conservative or invasive) based on evidence of whether the intervention improves OHRQoL [14, 15]. Determination of MID is challenging and several methods are suggested [15]. While some researchers used the anchorage method to establish the MID points for specific intervention on OHRQoL measure [16], Tsakos et al*.* [17] suggested calculating the effect size (ES) of the mean difference between groups of disease/condition status in cross-sectional data to statistically estimate the MID of the associations with OHRQoL measures. The use of effect size to estimate MID is argued to be less biased and makes it easy to compare between studies with different outcome measures or measures of exposure [15]. Several measures of standardized effect size are available however, Cohen’s *f*^2^ is argued to be a useful measure of effect size estimation considering it could estimate the effect size from multivariable hierarchical models [18] which account for potential confounders. Among the important confounders for OHRQoL such as biomedical and socioeconomic factors that needed to be accounted for when examining the association between TMD and OHRQoL, there has been a demand to include psychological factors [2] in assessing such a relationship.

Perceived stress, which refers to the extent an individual feels stressed with life events, is an important psychological factor that affects some aspects of oral health status. For instance, perceived stress is associated with recurrent aphthous ulcer [19], mechanical wear of teeth due to bruxism [20], reporting of dry mouth [21], poor perceived oral health [22], and perceived (work and non-work related) stress [1, 23]. While these variable associations are recognised in the literature, the earlier quantifications did not account for sampling biases adequately due to the sampling restrictions in those studies included in Dahlström and Carlsson [2] systematic review. National studies with representative samples can play an important role in providing for such quantification and help triangulate the association of TMD with OHRQoL. Thus, this study aimed to quantify the association between TMD experience and OHRQoL domains and overall measurement scores while accounting for various confounders as well as estimating the effects sizes for TMD experience association with OHRQoL measurement score for necessary across-study comparisons and statistical estimation of Minimally Important Difference (MID-S) for the association between TMD and OHIP-14.

## Methods

### Study design

We used the National Survey of Adult Oral Health (NSAOH) 2004–2006 wave [24] which is a cross-sectional three staged random stratified clustered representative sample of Australia’s adult residents aged 15-years or over who resided in a household with access to a telephone line that was listed in Australia’s “Electronic White Pages” [25] with their corresponding postcode. The first sampling stage selected the postcodes followed by the second sampling stage where households were selected from the selected postcodes. The third sampling stage selected the target person from the selected household. Stratification of the selected postcodes from all Australian states and territories was performed and included two strata: capital city and rest of the state, however, the Australian Capital Territory (ACT) was considered as a single stratum major city. Further details of NSAOH sampling are reported in Slade et al*.* [26]. The NSAOH data used in this study comprised three datasets: Computer Assisted Telephone Interview (CATI), Self-Complete Questionnaire and a Standardized Oral Epidemiological Examination.

### Setting

Australia’s residents in all states and territories aged 15-years or over who randomly invited to join the NSAOH. NSAOH data were collected between July 2004 and September 2006.

### Participants

The randomly selected adult aged 15-years or older present in the household at the time of the CATI interview was invited to join the study to proceed with the interview and if declined, it was reported as a declined response. Participants who completed the CATI were asked if they were happy to receive a mailed questionnaire and participate in the oral epidemiological examination. Participants who agreed to complete other parts of the study were contacted later for the mailed questionnaire and the oral epidemiological examination. For the oral epidemiological examination, participants had a medical questionnaire to determine their eligibility for the epidemiological oral examination according to the adopted protocol [27] such as rheumatic fever, endocarditis, bleeding disorders, joint replacement within the past 3-months, etc.

### Sample size, response rate and quality check

The target sample size of the NSAOH was calculated for key outcomes such as the Decayed, Missing and Filled teeth to detect, on a national level in the age-specific estimates, 10% reduction on the national level compared with 1988 survey at 80% power and was 7500 participants. Considering that TMD was not one of the outcomes considered for sample size calculation, we provided posthoc power analysis. The average response rate for the NSAOH 2004–06 by the selected postcode was 49.0% for the interview and 44% for the in-scope oral examination [26]. The non-response bias was investigated using the "population benchmark" and "small area socio-economic indicators" approaches and found to be small (less than 3%) for most oral health indicators [26]. Missing data were examined for key variables and was small (0–4.4%) which found to be at random and were not imputed. NSAOH data were weighted to account for sampling design and to match the age and gender distribution for the selected strata which were provided by the 2005 Australian Bureau of Statistics Estimated Residential Population Data [26]. Participants with complete records across the three datasets were included in this study.

### Ethical considerations

Ethical approval for the NSAOH was obtained from the University of Adelaide Human Research Ethics Committee with approval number: H-001-2004. Informed consent was obtained from all NSAOH participants. For the CATI interview, a verbal consent was obtained from the participants before proceeding with the CATI interview due to the nature of the survey method, and was included in the ethics approval. Written consent was obtained from participant for the self-complete questionnaire and the standardized oral epidemiological examination. Participants found on their oral examination to have a concerning medical or dental condition were referred appropriately to health professionals.

### NSAOH dataset and variables

#### Self-complete questionnaire

OHRQoL (outcome variable) measured by the Oral Health Impact Profile—Short form (OHIP-14)

The mailed self-completed questionnaire collected data on OHRQoL using the 14-item Oral Health Impact Profile—short-form (OHIP-14) [28], a shorter version of the original OHIP-49. The OHIP-14 consists of seven domains with two items that represent each domain. OHIP-14 domains include functional limitation, physical pain, psychological discomfort, physical disability, psychological disability, social disability and handicap. Items for the OHIP-14 are scored on a 5-point scale ranging from 0 for "Never" to 4 for "Very often". The reference period of the OHIP-14 is "over the past 12 months". The total OHIP-14 scores range from 0 to 56 with a higher score meaning a lower OHRQoL. OHIP-14 was the most commonly used generic measure for oral conditions associated with OHRQoL with evidence of reliability and validity across a wide range of socio-cultural contexts [29].

### TMD experience (main exposure variable) measured by the Diagnostic Criteria Question for TMD

**Fig. 1:**
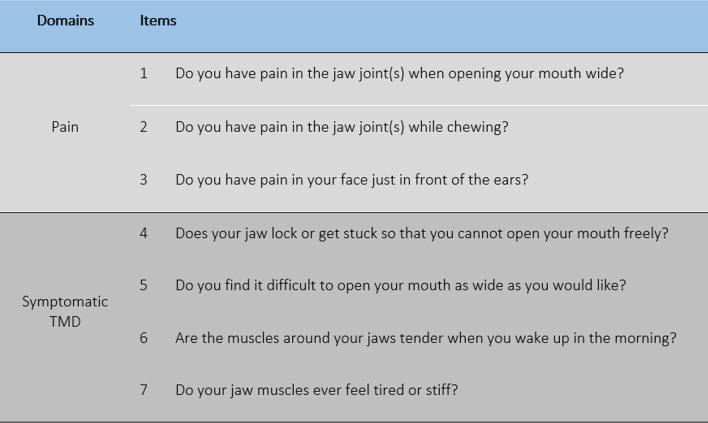
Diagnostic criteria question for TMD

TMD experience was assessed using the Diagnostic Criteria Question for TMD, which was adapted from a Canadian study where the questionnaire is reported to have 73% sensitivity and 75% specificity in predicting clinical diagnosis for TMD [30]. The questionnaire (Fig. 1) consists of seven items in two domains: pain (three questions) and symptomatic TMD (four questions) which represent functional disturbance. The criterion adopted for the presence of TMD was recording a positive response for one or more of the pain items and a positive response in one or more of the symptomatic TMD items which are in line with the clinical diagnosis of TMD used in previous research [31]. This research diagnostic criteria have been recommended by Sanders and Slade [32] and was used by researchers in a recent study [1].

### Perceived stress measured by the 14-item Perceived Stress Scale (PSS-14) (confounder variable)

The Perceived Stress Scale (PSS-14) was developed by Cohen et al*.* [33] as a measure of subjective stress and consists of 14 items representing how unpredictable, uncontrollable, and overloaded participants feel in their life. The reference period of the PSS-14 is”last month”. Items for the PSS-14 are scored on a 5-point Likert-like scales ranging from 0 for ‘Never’ to 4 for ‘Very often’. The PSS-14 consists of two subscales: Perceived distress (negative subscale) and Perceived control (positive subscale). The perceived distress subscale is the sum score for items 1,2,3,8,11,12 and 14 where higher scores represent higher perceived distress. The perceived control subscale is the sum score of items 4,5,6,7,9,10 and 13 where higher score represent higher perceived control (coping).

### Self-reported static malocclusion (confounder)

The NSAOH self-complete questionnaire asked the participants whether their ‘Teeth feel do not fit properly together’ whereas the response was yes/no. We have used this question as a proxy of static malocclusion.

### Alcohol consumption (confounder)

The NSAOH participants were asked about the 'Days per week of alcohol drinking' and 'The number of standard drinks per day'.

#### The Computer Assisted Telephone Interview (CATI) data

The CATI collected demographic and socioeconomic characteristics (included as confounders or predictors in our analysis) such as age, gender, educational attainment, current employment status, annual household income, having private dental insurance, indigenous status and country of birth. Further, NSAOH collected information on chronic diseases experience such as diabetes and behaviours such as current smoking status.

#### Standardized oral epidemiological examination data

The standardized dental examinations were carried out by 30 dentists who received training to comply with the oral examination protocol [34]. The intra-examiner reliability was checked for five participants for each dentist with replicated examinations conducted by the principal survey examiner for teeth present, caries experience and periodontitis. The median inter-examiner reliability was 0.85. The oral examination was carried out using a self-illuminated dental mirror and a periodontal probe (with 2 mm marking). The oral examination included:

### Number of missing teeth (confounder)

The number of missing permanent teeth for any reason and not replaced by a removable or fixed prosthesis was recorded starting from the upper right quadrant at the position of tooth 18 to 28 then the lower left quadrant at tooth position 38 to 48.

### Number of teeth with untreated decay (confounder)

The NSAOH examiners visually inspected (using the self-illuminated dental mirror and periodontal probe gently used to check the surface texture) the five surfaces of the present permanent teeth (occlusal, mesial, distal, buccal and lingual) for decay in enamel, dentine or involved enamel and dentine and recorded as ‘D’. Recording of teeth with untreated decay started from tooth 18 if present to 28 then 38 to 48. The number of teeth with untreated decay was computed by summing the number of present teeth with untreated decay lesion on one or more of the examined surfaces.

### Number of filled teeth (confounder)

The oral examiners visually inspected tooth surfaces for fillings using the same procedure for recording decayed teeth. The number of filled teeth was computed by summing the number of present teeth with one filling or more.

### Periodontal diseases (predictor)

Participants of the NSAOH were examined for recession and probing depth which were used to compute clinical attachment loss (CAL). Measurements were obtained from three points: mesiobuccal, mid-buccal and distobuccal for all teeth present except for third molars. Periodontitis status was assessed using the US Centre for Disease Control and Prevention case definition of periodontitis [35]. For this study, we created a binary variable (Yes/No) for the presence of ‘moderate or severe periodontitis’ where moderate periodontitis was defined as the presence of ≥ 2 sites of inter-proximal CAL of ≥ 4 mm, and severe periodontitis was defined as the presence of ≥ 2 sites of inter-proximal CAL of ≥ 6 mm.

### Data analysis plan

Data were analysed using SPSS Statistics for Windows version 27 [36] and STATA 14 IC [37]. We conducted a Confirmatory Factor Analysis (CFA) to check the structural validity of the used DCQ for TMD using the generalized structural equation model (Additional file 1: Figure S1 and Table S1 represented CFA of the two-dimensional DCQ for TMD model, and Figure S2 and Table S2 represented the CFA for the one-dimensional DCQ for TMD model). We found that the two-dimensional structure of the DCQ for TMD had slightly better goodness of fit statistics compared with the one-dimensional structure. To account for the sampling weights, strata and clusters of the NSAOH, a complex sample analysis module in SPSS were used in all conducted analyses [38]. The characteristics of the participants were obtained using complex sample descriptive statistics using percentages or mean of population estimates with a 95% confidence interval as well as a distribution analysis of OHIP-14 scores. To explore factors associated with TMD experience as an exposure, we conducted bivariate associations with potential confounders using complex sample bivariate logistic regression models. Further, we conducted complex samples Independent Sample *t*-test for OHIP-14 domains and total scores and TMD experience as well as conducted a posthoc power analysis. Moreover, we computed Cronbach's alpha reliability for the used scales (OHIP-14 and PSS-14 subscales). We used adjusted multivariate complex samples linear regression models for OHIP-14 domains and total scores for the association with TMD experience. To examine the association between TMD experience and OHIP-14 scores while accounting for various confounders, multivariable block-wise complex sample linear regression models for OHIP-14 scores were conducted using consecutive blocks. Block 1: TMD experience, Block 2: Block 1 + components of oral health status (Moderate or severe periodontitis, number of decayed, missing and filled teeth, and whether teeth are not fit together properly), Block 3: Block 2 + demographics (sex, age group, birthplace and Indigenous status), Block 4: Block 3 + socioeconomics (educational attainment, current employment status, annual household income level and having private dental insurance) and Block 5: Block 4 + health and health behaviours (diabetes status, perceived stress subscales, current smoking status and alcohol consumption). To estimate the minimally important difference statistically (MID-S) of TMD association with OHIP-14 scores, we used Cohen’s *f*^2^ as an appropriate measure of effect size [18, 39]. The Cohen’s *f*^2^ was calculated using the following equation: *f*^2^ = *R*^2^_AB_ − *R*^2^_A_/1 − R^2^_AB_ [18] where R^2^_AB_ was the OHIP-14 variance explained in multivariable linear regression by TMD experience together with a set of confounders and *R*^2^_A_ is the OHIP-14 variance explained by confounders in the multivariable linear regression (excluding the TMD experience). We used Cohen’s *f*^2^ index to determine the magnitude of the effect size where *f*^*2*^ ≥ *0.02, f*^*2*^ ≥ *0.15, and f*^*2*^ ≥ *0.35* represent small, medium, and large effect sizes, respectively [18, 39]. Supplementary analyses were presented in the Additional file 1: Supplementary analyses that included a confirmatory factor analysis for the DCQ for TMD, examination of regression residuals and an alternative complex samples linear regression models for log-transformed OHIP-14 overall and domains scores and included a confirmatory factor analysis for the DCQ for TMD, examination of regression residuals and an alternative complex samples linear regression models for log-transformed OHIP-14 overall and domains scores.

## Results

### NSAOH participants characteristics

A total of 4133 Australian adults completed all three parts of the NSAOH and were included in our analyses. Sample characteristics are shown in Table 1. The most common age group was 35–44 years 19.7% (95% CI: 17.9–21.6%) followed by 25–34 years 19.1% (95% CI: 16.5–22.0%).There was a higher proportion of those with secondary school or less educational attainment 40.3% (95% CI: 37.6–43.1%) and those who were currently employed 65.1% (95% CI: 62.4–67.7%). A minority of the participants were born overseas (21.0%, 95% CI: 18.9–23.2%), and about half of the sample were living in a household earning less than $60,000 annually (53.1%, 95% CI: 49.8–56.4%). TMD experience prevalence was 9.9% (95% CI: 8.4–11.6%), while moderate to severe periodontitis was prevalent in 21.4% (95% CI: 19.5–23.4%) of participating adults. The mean number of decayed teeth was 0.6 (95% CI: 0.5–0.7), missing teeth was 5.7 (95% CI: 5.5–6.0) and filled teeth was 7.9 (95% CI: 7.5–8.2). The perceived distress subscale showed high reliability (Cronbach's Alpha = .0.9) and the mean perceived distress subscale score was 12.1 (95% CI: 11.8–12.3) while the perceived control subscale showed high reliability (Cronbach's Alpha = 0.8) and the mean perceived control score was 17.6 (95% CI: 17.3–17.8). The OHIP-14 scores skewness was 1.6 (SE = 0.03) and kurtosis was 2.8 (SE = 0.07) which were considered within the acceptable normal distribution as argued by Watson [40] if skewness is between ‐2 to + 2 and kurtosis is between − 7 to + 7. Further, OHIP-14 scores showed high reliability (Cronbach's Alpha = 0.9) whereas the mean OHIP-14 score was 7.12 (95% CI: 6.69–7.54). Moreover, we explored factors associated with TMD experience using bi-variate associations as shown in Table 1.

**Table 1:** Participants characteristics and complex sample bi-variate association with TMD experience

Parameter	NSAOH participants (*n* = 4133)	TMD (yes)			
	% (95% CI of %)	% of total (95% CI) 9.9% (95% CI: 8.4–11.6))	Bi-variate association		
			*OR*	95% *CI* of *OR*	*P* value^1^
*Age group*					
15–24 years	18.1 (15.4–21.2)	2.1 (1.2–3.7)	3.0	1.5–6.3	.003
25–34 years	19.1 (16.5–22.0)	2.4 (1.7–3.5)	3.4	1.9–6.0	< .001
35–44 years	19.7 (17.9–21.6)	2.2 (1.7–2.8)	2.9	1.8–4.7	< .001
45–54 years	17.6 (16.0–19.3)	1.6 (1.2–2.3)	2.4	1.4–4.1	.002
55–64 years	12.9 (11.6–14.3)	1.0 (0.7–1.3)	1.9	1.2–3.2	.008
≥ 65 years	12.5 (11.3–13.9)	0.5 (0.3–0.8)	Ref.	–	–
Sex (female)	50.0 (47.4–52.7)	6.4 (5.4–7.6)	2.0	1.3–3.0	.002
*Educational attainment*					
University qualification	32.7 (30.0–35.6)	2.5 (1.9–3.2)	0.7	0.4–1.0	.062
Vocational education	27.0 (24.6–29.5)	3.2 (2.3–4.5)	1.1	0.7–1.8	.599
Secondary school or less	40.3 (37.6–43.1)	4.2 (3.2–5.5)	Ref.	–	–
Currently employed (yes)	65.1 (62.4–67.7)	6.1 (5.0–7.6)	0.9	0.6–1.2	.392
*Annual housed income*					
Less than $60 k	53.1 (49.8–56.4)	5.5 (4.6–6.5)	1.3	0.9–1.9	.131
$60 k or more	46.9 (43.6–50.2)	3.7 (2.7–5.1)	Ref.	–	–
Birth place (overseas)	21.0 (18.9–23.2)	1.9 (1.4–2.7)	0.9	0.6–1.4	.680
Have private dental insurance (yes)	47.3 (44.4–50.2)	3.8 (3.0–4.9)	0.7	0.5–0.9	.018
Diabetic (yes)	4.3 (3.2–5.6)	0.4 (0.2–0.8)	1.0	0.5–2.0	.996
Indigenous Australian (no)	98.9 (98.3–99.4)	9.7 (8.3–11.4)	0.7	0.2–2.3	.568
Current smoker (yes)	15.0 (13.1–17.1)	2.4 (1.6–3.5)	2.0	(1.2–3.1)	.005
Moderate/severe periodontitis (yes)	21.4 (19.5–23.4)	1.8 (1.3–2.5)	0.8	0.6–1.3	.381
Teeth feel do not fit properly together (yes)	21.0 (18.6–23.5)	5.8 (4.6–7.5)	3.0	2.1–4.3	< .001

	Mean (95% *CI*)	Mean (95% *CI*)	*OR*	95% CI of *OR*	*P* value^1^
No. of decayed teeth	0.6 (0.5–0.7)	1.0 (0.51.5)	1.2	1.0–1.3	.006
No. of missing teeth	5.7 (5.5–6.0)	5.4 (4.8–6.0)	0.99	0.96–1.01	.261
No. of filled teeth	7.9 (7.5–8.2)	7.5 (6.4–8.6)	0.99	0.96–1.02	.413
*Alcohol consumption*					
Days per week of alcohol drinking	2.5 (2.4–2.6)	2.0 (1.6–2.4)	.89	.81–.99	.037
No. of standard drinks per day	2.4 (.1–2.3)	2.6 (2.0–3.2)	1.01	.94–1.11	.670
*PSS-14 subscales*	–	–	–	–	–
Perceived distress	12.1 (11.8–12.3)	14.2 (13.1–15.3)	1.10	1.05–1.16	< .001
Perceived control	17.6 (17.3–17.8)	16.3 (15.3–17.3)	.94	.90–.98	.003

Analyses accounted for cluster and stratum used in NSAOH sampling strategy, as well as sampling weights to ensure representativeness of the estimates

*OR*: odds ratio of bi-variate association, *CI*: confidence interval, OHIP-14: Oral Health Impact Profile (short form), PSS-14: the 14-item Perceived Stress Scale, Ref.: Reference category

^1^Bi-variate complex samples logistic regression model for TMD experience

### TMD experience association with OHIP-14 domains and total scores

On assessing the OHIP-14 domains impaired by TMD experience after adjusting for confounders, TMD experience was associated with impairments in all OHIP-14 domains (*P* < 0.05) where higher impairments were observed in the physical pain (*B* = 0.82, 95% CI: 0.45–1.20, *P* < 0.001) with small effect size (*f*^2^ = 0.022), psychological discomfort (*B* = 0.68, 95% CI: 0.29–1.06, *P* = 0.001) and psychological disability (*B* = 0.52, 95% CI: 0.20-0.84, *P* = 0.001) domains of OHIP-14 as shown in Table 2. The difference in the mean OHIP-14 scores for those reporting TMD (Mean = 13.1, SD = 9.50, 95% CI: 12.0–14.0) and those who did not (Mean = 6.6, SD = 7.044, 95% CI: 6.0–6.8) was statistically significant (*t* = 7.51, *P* < 0.001) (Table 2) with 100% observed power. In the unadjusted complex sample linear regression model for OHIP-14 scores (Table 3, Block 1), TMD experience was associated with higher OHIP-14 scores (*B* = 5.95, 95% CI: 4.39–7.50, *P* < 0.001) whereas Cohen’s *F*^2^ was 0.058. On adding the other oral health factors (Table 3, Block 2) to the complex sample linear regression model for OHIP-14 scores, TMD experience was associated with higher OHIP-14 scores (*B* = 4.17, 95% CI: 2.77–5.56, *P* < 0.001) where Cohen’s *F*^2^ of TMD association was 0.032. Similarly, when we added the demographics (Table 3,Block 3) to the model, TMD experience was associated with higher OHIP-14 scores (*B* = 3.70, 95% CI: 2.31–5.10, *P* < 0.001) where Cohen’s *F*^2^ of TMD association was 0.026 and when adding the socio-economic characteristics (Table 3,Block 4), TMD experience was associated with higher OHIP-14 score (*B* = 3.64, 95% CI: 2.07–5.21, *P* < 0.001) where Cohen’s *F*^2^ of TMD association was 0.024. In the fully-adjusted multivariable models for OHIP-14 scores (Table 3, Block 5), TMD experience was associated with higher OHIP-14 scores (*B* = 3.34, 95% CI: 1.94–4.75, *P* < 0.001) where the Cohen’s *f*^2^ effect size of TMD association with OHIP-14 scores was 0.022. Furthermore, we observed in the fully adjusted model for OHIP-14 scores that the perceived distress subscale (*B* = 0.38, 95% CI: 0.28–0.47, *P *< 0.001) and the perceived control subscale (*B* = -0.10, 95% CI: − 0.18–0.02, *P* = 0.012) of the PSS-14 were associated with OHIP-14 scores. We examined the regression standardized residuals histogram which appeared to have an acceptable normal distribution (Additional file 1: Figure S3).

**Table 2:** OHIP-14 domains and total scores among the Australians and associations with TMD experience status

OHIP-14 domains	Population estimate	Independent sample t-test for complex samples of OHIP-14 domains and total score by TMD experience status^1^					Adjusted multivariate complex samples linear regression models for OHIP-14 domains and total score associations with TMD experience status^1,2,3^			
		TMD (Yes)	TMD (no)							
	Mean (95% *CI*)	Mean (95% *CI*)	Mean (95% *CI*)	*t*	*P* value	*B*	SE	95% *CI* of *B*	*P* value	*f*^*2*^
1 Functional limitation	.57 (.51–.63)	1.05 (.86–1.24)	.52 (.46–.58)	5.24	< .001	.36	0.12	.13–.59	.002	0.001
2 Physical pain	2.09 (2.00–2.19)	3.16 (2.80–3.52)	1.98 (1.88–2.07)	6.37	< .001	.82	0.19	.45–1.20	< .001	0.022
3 Psychological discomfort	1.62 (1.51–1.72)	2.81 (2.48–3.15)	1.49 (1.38–1.60)	7.36	< 001	.68	0.19	.29–1.06	.001	0.012
4 Physical disability	.66 (.60–.72)	1.27 (1.01–1.53)	.60 (.54–.66)	4.97	< .001	.33	0.14	.06–.60	.016	0.007
5 Psychological disability	1.11 (1.04–1.19)	2.09 (1.79–2.39)	1.01 (.93–1.09)	6.92	< 001	.52	0.16	.20–.84	.001	0.012
6 Social disability	.55 (.49–.60)	1.11 (.85–1.36)	.48 (.43–.54)	4.81	< .001	.32	0.10	.12–.51	.002	0.008
7 Handicap	.51 (.45–.57)	1.00 (.77–1.23)	.46 (.40–.52)	4.51	< .001	.27	0.10	.07–.47	.009	0.007
OHIP-14 scores	7.12 (6.69–7.54)	12.48 (10.96–13.99)	6.53 (6.09–6.96)	7.51	< .001	3.34	0.72	1.94–4.75	< .001	0.022

^1^Analyses accounted for cluster and stratum used in NSAOH sampling strategy, as well as sampling weights to ensure representativeness of the estimates

^2^Multivariate complex samples linear regression models for OHIP-14 domains and total score association with TMD experience status adjusted for oral health status (Moderate or severe periodontitis, number of decayed, missing and filled teeth, and whether teeth are not fit together properly), demographics (sex, age group, birthplace and Indigenous status), socioeconomics (educational attainment, current employment status, annual household income level and having private dental insurance) and health and health behaviours (diabetes status, perceived stress subscales, current smoking status. and alcohol consumption)

^3^The provided *f*^2^ is for TMD association with OHIP-14 domains and total score using the equation presented in the methods section

**Table 3:** Multivariable block-wise complex sample linear regression models for OHIP-14 score among Australian adults

	Block 1			Block 2			Block 3			Block 4			Block 5		
	*B*	95% *CI* of *B*	*P* value	*B*	95% *CI* of *B*	*P* value	B	95% *CI* of *B*	*P* value	*B*	95% *CI* of *B*	*P* Value	*B*	95% *CI* of *B*	*P* Value
TMD (yes)	5.95	4.39–7.50	5.95	4.17	2.77–5.56	< .001	3.70	2.31–5.10	< .001	3.64	2.07–5.21	< .001	3.34	1.94–4.75	< .001
Moderate/severe periodontitis (yes)	–	–	–	0.98	0.06–1.90	.036	1.43	0.53–2.32	.002	1.45	0.55–2.34	< .001	1.38	0.52–2.23	.002
Teeth feel don’t fit properly together (yes)	–	–	–	4.75	3.80–5.70	< .001	4.59	3.66–5.52	< .001	4.29	3.36–5.22	< .001	3.57	2.67–4.47	< .001
No. of decayed teeth	–	–	–	1.17	0.72–1.62	< .001	1.16	0.70–1.61	< .001	1.03	0.51–1.54	< .001	0.95	0.46–1.43	< .001
No. of missing teeth	–	–	–	0.17	0.11–0.22	< .001	0.31	0.23–0.38	< .001	0.28	0.20–0.37	< .001	0.29	0.21–0.37	< .001
No. of filled teeth	–	–	–	0.07	0.02–0.13	.005	0.16	0.09–0.23	< .001	0.15	0.08–0.21	< .001	0.15	0.09–0.22	< .001
Sex (female)	–	–	–	–	–	–	0.93	0.11–1.75	.026	0.74	–0.13–1.62	.096	.41	–.47–1.29	.361
*Age group*	–	–	–	–	–	–	–	–	–	–	–	–	–	–	–
15–24 years	–	–	–	–	–	–	5.01	3.14–6.88	< .001	5.08	2.81–7.34	< .001	3.44	1.39–5.49	.001
25–34 years	–	–	–	–	–	–	4.54	2.92–6.16	< .001	4.74	2.99–6.49	< .001	3.37	1.75–4.99	< .001
35–44 years	–	–	–	–	–	–	4.44	3.20–5.69	< .001	4.71	3.32–6.11	< .001	3.35	2.04–4.66	< .001
45–54 years	–	–	–	–	–	–	3.58	2.50–4.65	< .001	4.04	2.73–5.35	< .001	2.73	1.52–3.95	< .001
55–64 years	–	–	–	–	–	–	2.30	1.23–3.37	< .001	2.62	1.42–3.81	< .001	1.82	0.69–2.94	.002
≥ 65 years	–	–	–	–	–	–	Ref.	–	–	Ref.	–	–	Ref.	–	–
Birth place (overseas)	–	–	–	–	–	–	1.50	0.60–2.39	.001	1.37	0.53–2.21	.002	1.50	0.67–2.32	< .001
Indigenous Australian (no)	–	–	–	–	–	–	–1.76	− 4.54–1.02	.214	− 1.48	− 4.93–1.96	.398	− 1.05	− 4.25–2.14	.517
*Educational attainment*	–	–	–	–	–	–	–	–	–	–	–	–	–	–	–
University qualification	–	–	–	–	–	–	–	–	–	− 0.55	− 1.39–0.28	.192	− 0.70	− 1.55–0.16	.110
Vocational education	–	–	–	–	–	–	–	–	–	0.28	− 0.66–1.22	.556	0.26	− 0.65–1.17	.574
Secondary school or less	–	–	–	–	–	–	–	–	–	Ref.	–	–	Ref.	–	–
Currently employed (yes)	–	–	–	–	–	–	–	–	–	0.27	− 0.69–1.23	.580	0.25	− 0.66–1.17	.587
*Annual household income*	–	–	–	–	–	–	–	–	–	–	–	–	–	–	–
Less than $60 k	–	–	–	–	–	–	–	–	–	1.43	0.51–2.35	.002	1.32	0.45–2.19	.003
$60 k or more	–	–	–	–	–	–	–	–	–	Ref.	–	–	Ref.	–	–
Have private dental insurance (yes)	–	–	–	–	–	–	–	–	–	− 0.62	− 1.47–0.23	.150	− 0.61	− 1.37–0.16	.119
Diabetic (yes)	–	–	–	–	–	–	–	–	–	–	–	–	0.80	− 1.75–3.35	.538
Currently a smoker (yes)	–	–	–	–	–	–	–	–	–	–	–	–	− 0.33	− 1.56–0.91	.603
*Alcohol consumption*	–	–	–	–	–	–	–	–	–	–	–	–	–	–	–
Days per week of alcohol drinking	–	–	–	–	–	–	–	–	–	–	–		− 0.04	− 0.20–0.12	.586
No. of standard drinks per day	–	–	–	–	–	–	–	–	–	–	–	–	0.11	− 0.07–0.29	.241
*PSS-14 subscales*	–	–	–	–	–	–	–	–	–	–	–	–	–	–	–
Perceived distress	–	–	–	–	–	–	–	–	–	–	–	–	0.38	0.28–0.47	< .001
Perceived control	–	–	–	–	–	–	–	–	–	–	–	–	− 0.10	− 0.18–0.02	.012
Model *R*^2^	.054			*R*^2^_AB_ = .192, *R*^2^_A_ = .165			*R*^2^_AB_ = .222, *R*^2^_A_ = .201			*R*^2^_AB_ = ., *R*^2^_A_ =			*R*^2^_AB_ = .296, *R*^2^_A_ = .281		

Analyses accounted for cluster and stratum used in NSAOH sampling strategy, as well as sampling weights to ensure representativeness of the estimates

*B*: estimate of linear regression coefficient, *CI*: confidence interval, OHIP-14: Oral Health Impact Profile (short form), PSS-14: the 14-item Perceived Stress Scale, model *R*^2^: proportion of OHIP-14 variance explained in the model, *R*^2^_A_: proportion of OHIP-14 variance explained in the model by all other explanatory variables excluding TMD experience*, R*^2^_AB_: proportion of OHIP-14 variance explained in the model by TMD experience and all other explanatory variables, Ref.: Reference category

## Discussion

In this study, we aimed to quantify, among a representative sample of Australian adults, the association between TMD and OHRQoL dimensions and overall measurement scores while accounting for the hierarchical structure of the data and the relevant confounding variables. We found that TMD experience was associated with impairments to all seven OHRQoL domains measured by the OHIP-14 with higher impairments observed in the physical pain, psychological discomfort and psychological disability domains of the OHIP-14. Furthermore, on examining the association between TMD experience and the overall OHRQoL measured by OHIP-14 scores using multivariate regression models, TMD had a significant negative association with OHRQoL and this association remained significant while accounting for various confounders. However, when we estimated the minimally important difference of the association between TMD experience and the OHRQoL measure statistically using a standardized estimate of effect size, it was found to be small. Moreover, we found that higher levels of perceived distress and perceived control were associated with TMD experience and OHRQoL suggesting a confounding effect.

The principal finding of this study was that TMD experience was associated with impairments to all OHRQoL domains measured by the OHIP-14 suggesting the broad extent to which TMD impaired the participant’s perception of their wellbeing. While it might not be surprising that TMD experience was associated with the physical pain domain [3] with a small clinical relevance observed, It was interesting to observe associations with psychological discomfort and psychological disability in relation to OHRQoL. This observation might explain why pharmacological treatment with non-steroidal anti-inflammatory drugs (NSAID) and selective serotonin reuptake inhibitors (SSRIs) showed effectiveness in improving OHRQoL in TMD patients [14]. Moreover, TMD experience was associated with poorer overall OHRQoL. This finding is consistent with smaller studies included in the Dahlström and Carlsson [2] and Oghli et al*.* [3] systematic reviews.

We attempted to estimate whether TMD experience was clinically relevant using a statistical approach to determine relevance for clinicians, health services providers and health funds to offer treatment and whether the intervention provided is effective [41]. While effect size of mean difference of OHIP-14 scores across groups of a disease or a condition in cross-sectional data is recommended by Tsakos et al*.* [17] as a statistical method to estimate MID, we argued that using Cohen’s *f*^2^ to estimate the effect size might provide a standardized effect size estimate of TMD experience association with OHIP-14 score while considering confounders for OHIP-14 scores. The standardized effect size estimate will enable researchers to compare our findings across studies and OHRQoL outcome measures for association with TMD experience as well as it is argued to be less biased [15]. When we assessed the effect size of TMD experience association with OHIP-14 scores, it appeared to be small suggesting a small MID-S. The observed small MID-S of TMD experience association with OHRQoL measure might be interpreted with caution considering the OHIP-14 is a generic OHRQoL measure and other condition-specific OHRQoL measures such as the OHIP-TMD might show a different MID-S [42]. Accordingly, our findings might support screening for TMD when providing regular dental care similar to the current practice for caries and periodontitis experiences. Further, health services researchers might need to consider TMD as a component of oral health status when conducting OHRQoL research.

In our study, we included the psychological factor of perceived stress as it might be associated with oral health and oral health perception [19–21]. We found that increased perceived distress and perceived control subscales of the perceived stress scale were associated with TMD experience and OHRQoL suggesting a confounding effect. While it might be argued that OHIP-14 and PSS-14 are measuring psychological aspects, the measured psychological constructs by the two instruments are different which reduced any potential of bias. Accordingly, future research investigating TMD association with OHRQoL might need to consider psychological factors such as perceived stress in the analysis similar to the current practice of including biomedical and socioeconomic factors.

One limitation of our study was the use of the diagnostic criteria question for TMD which varies from proposed TMD questions by the International Network of Orofacial Pain and Related Disorders Methodology [43]. Further, the NSAOH oral epidemiological examination protocol did not include clinical examination for TMD [34]. However, the used TMD questionnaire has been validated using clinical data in another study [30] and reported to have a high level of sensitivity and specificity with the clinical diagnosis of TMD [32] which encouraged its use in past studies [1, 32]. It is also pertinent to note that the perceived impairment found in the measure of TMD and OHRQoL gives credence to the presence of a real impairment felt by the individuals with TMD. Also, we acknowledged the temporality of the observed associations between TMD and OHRQoL given the cross-sectional design of the study. While we argued that OHIP-14 scores have an acceptable normal distribution [40] for using the linear regression model, close findings were obtained via an alternative modelling approach using the complex samples linear regression for log-transformed OHIP-14 overall and domains scores (Additional file 1: Table 3 and Table 4). Our study carried limitations associated with secondary data analysis which limited our ability to use a current case definition of periodontitis however, this has a limited effect on our finding as periodontitis surveillance was not the focus of this study. Further, the chronic medical conditions included in our analysis was limited to diabetes since data on other chronic medical conditions were not collected as part of the NSAOH such as osteoarthritis. However, this unlikely to impact our findings considering that osteoarthritis is common in the older age group and might only impact a limited subgroup of TMD patients.

This study adds to the knowledge in the field by identifying the negative association between TMD and OHRQoL domains and overall measurement scores using a representative sample of the Australian adult population. Further, our analysis has adjusted for a range of biomedical, psychological and socioeconomic confounders that might not be included in a single study due to the limitation of such a quality dataset which adds to the reliability of our findings. Our findings contributed to clinical practice by highlighting TMD relevance, as a component of oral health status, to OHRQoL—an important outcome for healthcare services. In addition, our study was able to explain the reported effectiveness of pharmacological treatment of TMD using NSAIDs and SSRIs in improving OHRQoL in TMD patients considering our findings of higher observed associations of TMD experience with physical pain, psychological discomfort and psychological disability domains of OHRQoL. Besides, this study has revealed the confounding effect of psychological factors measured by perceived stress subscales in the relationship between TMD experience and OHRQoL which might support its inclusion in future TMD and OHRQoL research.

## Conclusion

In a representative sample of the Australian adult population, TMD experience was associated with lower OHRQoL, and this association remained significant after accounting for biomedical, psychological, and socioeconomic confounders. This finding supports that clinicians and health services providers might need to consider TMD in the overall OHRQoL of patients receiving dental care since OHRQoL is an end outcome of the provided oral healthcare services. When we used the standardized effect size estimate to determine whether TMD experience is clinically relevant to our participants’ OHRQoL in an adjusted analysis, we found that it was of a small clinical relevance. Considering that we used a generic OHRQoL measure, clinicians might need to consider individual variation in the perception of how TMD experience might be associated with the patient’s OHRQoL during decision-making about the need to provide an intervention (conservative or invasive). Besides, we found that TMD experience was associated with impairments to all OHRQoL domains measured by the OHIP-14. Also, we found that higher impairments were observed in physical pain, psychological disability and psychological discomfort which might explain the reported effectiveness of NSAID and SSRIs in improving the OHRQoL in TMD patients. Further, we found that increased levels of perceived distress and perceived control subscales of the perceived stress scale were associated with TMD experience and OHRQoL suggesting a confounding effect that might support the inclusion of perceived stress in TMD and OHRQoL research similar to the practice with biomedical and socioeconomic confounders.

## Supplementary Information

**Additional file 1.** Supplementary analyses that included a confirmatory factor analysis for the DCQ for TMD, examination of regression residuals and an alternative complex samples linear regression models for log-transformed OHIP-14 overall and domains scores.

## Data Availability

The data analysis output file is available upon request from the Australian Research Centre for Population Oral Health, Adelaide Dental School, The University of Adelaide.

## Abbreviations

CATI: Computer-Assisted Telephone Interview
NSAOH: National Survey of Adult Oral Health
OHIP-14: Oral Health Impact Profile-Short Form
OHRQoL: Oral Health-related Quality of Life
PSS-14: 14-Item Perceived Stress Scale
QoL: Quality of Life
TMD: Temporomandibular dysfunction
