# Supplementary material for: Temporomandibular dysfunction experience is associated with oral health-related quality of life: an Australian national study
Source: BMC Oral Health. 2021 Sep 6;21:432. doi: 10.1186/s12903-021-01773-z (PMC8422667; doi:10.1186/s12903-021-01773-z)
Supplement: Supplementary file 1 — Additional file 1. Supplementary analyses that included a confirmatory factor analysis for the DCQ for TMD, examination of regression residuals and an alternative complex samples linear regression models for log-transformed OHIP-14 overall and domains scores. [file 12903_2021_1773_MOESM1_ESM.docx]

# Supplementary analyses

## Confirmatory Factor Analysis (CFA) for the Diagnostic Criteria Question for TMD carried using Generalized Structural Equation model

We conducted a Confirmatory Factor Analysis (CFA) in Stata 14 IC. Considering that the Diagnostic Criteria Question for TMD items were dichotomous (Yes/No) for the presence or absence of TMD-related orofacial pain and functional limitations simultaneously, a generalized structural equation model was applied using the binomial logit function. Accordingly, the analysis fitted the DCQ for TMD items into one dimensional and two dimensional models as shown in Figure S1 and S2. Therefore, we were able to compare the Goodness of Fit between the one-dimension and the two dimension structure of the DCQ for TMD using Akaike Information Criterion (AIC) and Bayesian Information Criterion (BIC) goodness of fit indexes (Table S1 and Table S2). We found that the two-dimensional structure of the DCQ for TMD had slightly better goodness of fit statistics compared with the one-dimensional model. Please note that the model diagnostics for CFA are limited to the comparative fit indices (AIC and BIC) , due to the use of generalized linear models required here.


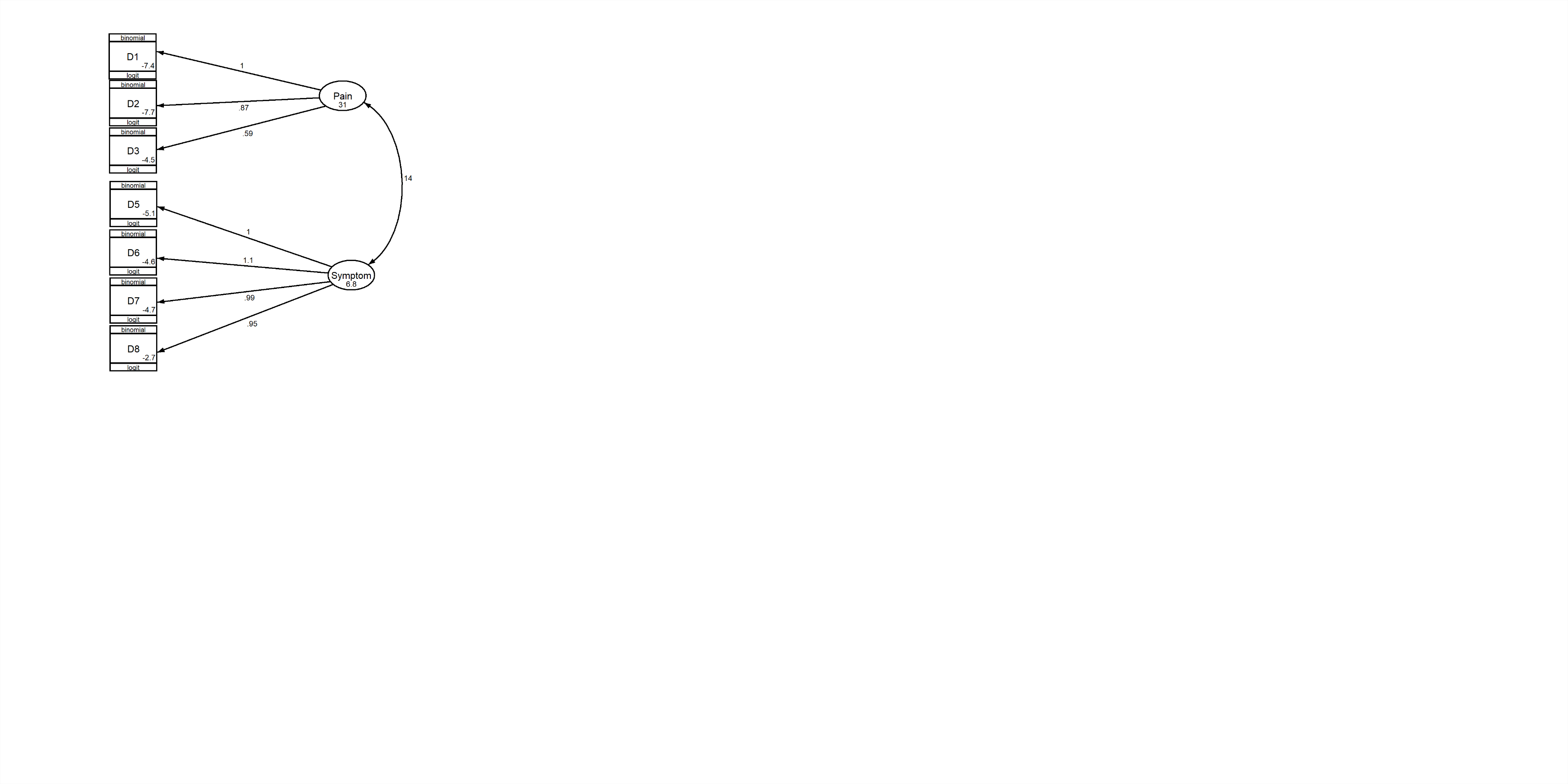


FigureS 1: Two-dimensional DCQ for TMD model

Table S1: Goodness of fit indices for the two-dimensional DCQ for TMD model.

| AIC | BIC |
| --- | --- |
| 12385.72 | 12480.64 |


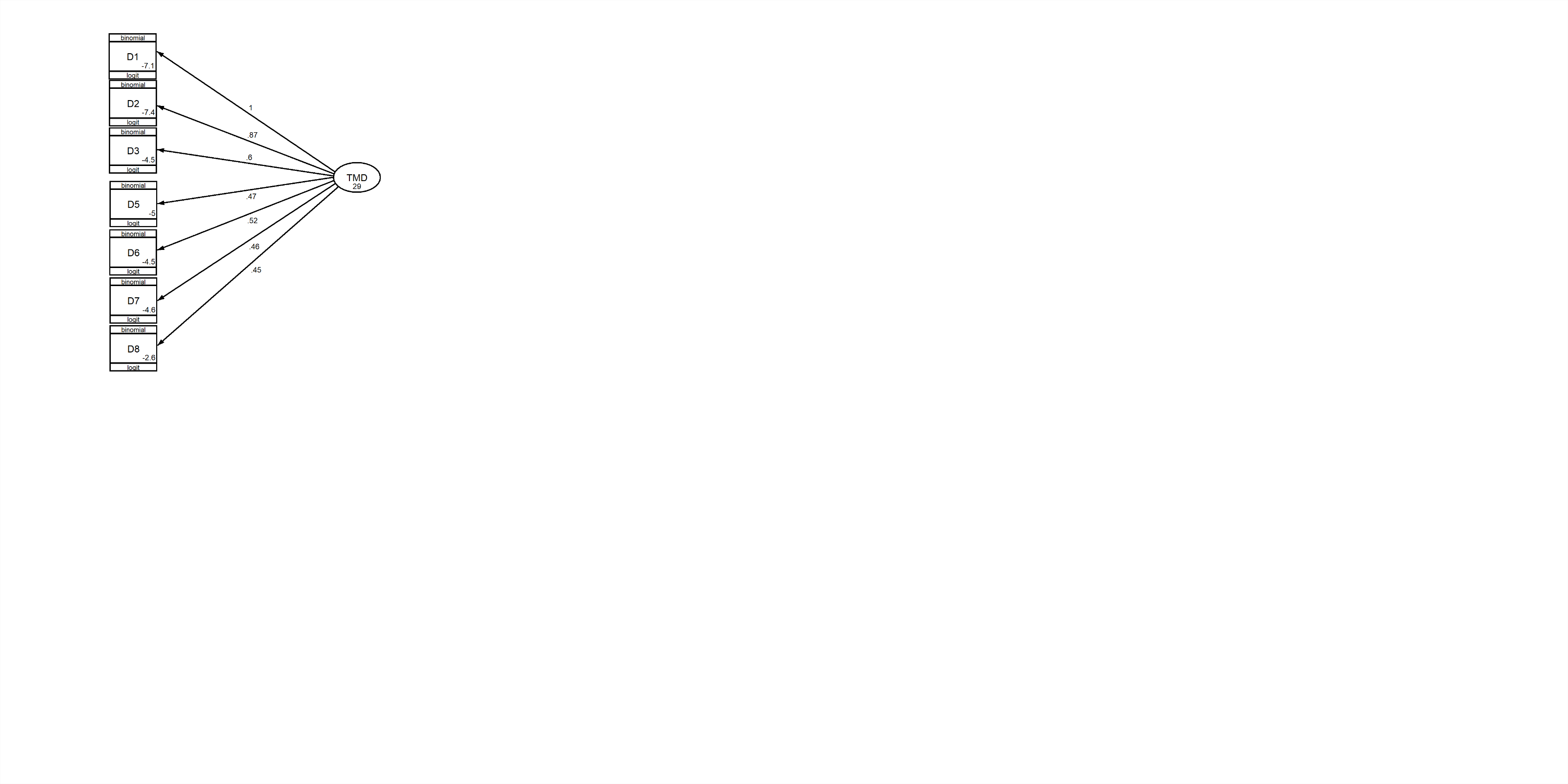


Figure S2: One-dimensional DCQ for TMD model

Table S2: Goodness of fit indices for the one-dimensional DCQ for TMD model.

| AIC | BIC |
| --- | --- |
| 12395.98 | 12484.58 |

## Supplementary analysis of regression residuals for the fully-adjusted complex samples linear regression model for OHIP-14 scores

We examined the distribution of the standardized regression residuals as shown on the residuals’ histogram (Figure S3) which showed an acceptable normality distribution.


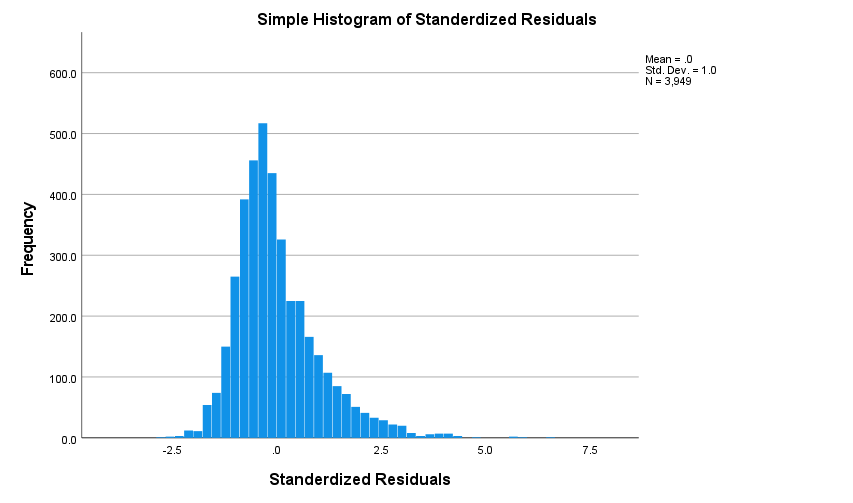


Figure S3: Histogram of standardized residuals of the fully-adjusted linear regression model for OHIP-14 overall scores

## Supplementary analysis of log-transformed OHIP-14 overall and domains scores

The distribution analysis of OHIP-14 scores revealed skewness of 1.6 (SE=0.03) and kurtosis of 2.8 (SE=.07) which we believe to be within the acceptable normal distribution [1] and therefore, the linear regression model we presented is a suitable modelling approach. To address concerns that might be raised regarding the skewness of OHIP-14 data, we have presented in this supplementary file an alternative modelling approach using log-transformed OHIP-14 overall and domains scores. On using the log-transformed OHIP-14 scores (Table S3), similar findings were observed in relation to the TMD association with OHIP-14 scores with a similar small effect size (*f*^2^=.02) as well as a similar confounding effect of the perceived stress subscale. The log-transformed OHIP-14 domains scores (Table S4) showed impairments associated with TMD experience with higher impairments observed in the physical pain, psychological discomfort and psychological disability.

Table S3: Multivariable complex sample linear regression models for log transformed OHIP-14 score among Australian adults*

|  | *B* | 95% *CI* of *B* | *P*-value |
| --- | --- | --- | --- |
| TMD (Yes) | 0.16 | 0.09-0.23 | <.001 |
| Moderate/severe periodontitis (Yes) | 0.05 | -0.01-0.11 | .086 |
| Teeth feel don’t fit properly together (Yes) | 0.19 | 0.14-0.24 | <.001 |
| No. of decayed teeth | 0.04 | 0.02-0.06 | <.001 |
| No. of missing teeth | 0.01 | 0.01-0.02 | <.001 |
| No. of filled teeth | 0.01 | 0.00-0.01 | <.001 |
| Sex (Female) | 0.02 | -0.03-0.07 | .473 |
| Age group | - | - | - |
| 15-24 years | 0.14 | 0.00-0.28 | .056 |
| 25-34 years | 0.13 | 0.03-0.24 | .014 |
| 35-44 years | 0.11 | 0.03-0.19 | .007 |
| 45-54 years | 0.10 | 0.02-0.17 | .010 |
| 55-64 years | 0.07 | 0.01-0.14 | .026 |
| ≥ 65 years | Ref. | - | - |
| Birth place (overseas) | 0.06 | 0.01-0.11 | .028 |
| Indigenous Australian (No) | -0.08 | -0.26-0.10 | .360 |
| Educational attainment | - | - | - |
| University qualification | -0.02 | -0.07-0.04 | .593 |
| Vocational education | 0.03 | -0.02-0.08 | .289 |
| Secondary school or less | Ref.- | - | - |
| Currently employed (Yes) | 0.03 | -0.03-0.08 | .371 |
| Annual household income | - | - | - |
| Less than $60k | 0.05 | -0.01-0.10 | .089 |
| $60k or more | Ref. | - | - |
| Have private dental insurance (Yes) | -0.04 | -0.09-0.01 | .121 |
| Diabetic (Yes) | 0.06 | -0.04-0.16 | .243 |
| Currently a smoker (Yes) | -0.02 | -0.09-0.05 | .653 |
| Alcohol consumption | - | - | - |
| Days per week of alcohol drinking | 0.00 | -0.01-0.01 | .543 |
| No. of standard drinks per day | 0.00 | -0.01-0.01 | .964 |
| PSS-14 subscales | - | - | - |
| Perceived distress | 0.02 | 0.01-0.02 | <.001 |
| Perceived control | -0.01 | -0.02-0.00 | .001 |
| Model *R*^2^ and *f*^2^ for TMD | *R*^2^_AB_=.231, *R*^2^_A_=.219, *f*^2^=.02 | | |

*Analyses accounted for cluster and stratum used in NSAOH sampling strategy, as well as sampling weights to ensure representativeness of the estimates.

Abbreviations: *B*: estimate of linear regression coefficient, *CI*: confidence interval, OHIP-14: Oral Health Impact Profile (short form), PSS-14: the 14-item Perceived Stress Scale, model *R*^2^: proportion of log transformed OHIP-14 variance explained in the model, *R*^2^_A_: proportion of log transformed OHIP-14 variance explained in the model by all other explanatory variables excluding TMD experience *,*  *R*^2^_AB_: proportion of OHIP-14 variance explained in the model by TMD experience and all other explanatory variables, Ref.: Reference category.

Table S4: Adjusted multivariate complex samples linear regression models for log-transformed OHIP-14 domains associations with TMD experience status^1,2,3^

|  | *B* | SE | 95% *CI* of *B* | *P*-value | *f^2^* |
| --- | --- | --- | --- | --- | --- |
| Functional limitation | .051 | .026 | -.001-.103 | .052 | .008 |
| Physical Pain | .081 | .022 | .037-.124 | <.001 | .013 |
| Psychological Discomfort | .077 | .023 | .032-.122 | .001 | .010 |
| Physical Disability | .039 | .031 | -.023-.101 | .220 | .004 |
| Psychological Disability | .056 | .024 | .008-.104 | .022 | .006 |
| Social Disability | -.013 | .028 | -.068-.042 | .649 | .002 |
| Handicap | .006 | .026 | -.046-.058 | .809 | .001 |

^1^ Analyses accounted for cluster and stratum used in NSAOH sampling strategy, as well as sampling weights to ensure representativeness of the estimates.

^2^ Multivariate complex samples linear regression models for OHIP-14 domains and total score association with TMD experience status adjusted for oral health status (Moderate or severe periodontitis, number of decayed, missing and filled teeth, and whether teeth are not fit together properly), demographics (sex, age group, birthplace and Indigenous status), socioeconomics (educational attainment, current employment status, annual household income level and having private dental insurance) and health and health behaviours (diabetes status, perceived stress subscales, current smoking status. and alcohol consumption).

^3^ The provided *f*^2^ is for TMD association with log-transformed OHIP-14 domains score using the equation presented in the methods section

References

1. Testing normality including skewness and kurtosis [<https://imaging.mrc-cbu.cam.ac.uk/statswiki/FAQ/Simon>]
